# Precision Medicine for Electrocardiogram Interpretation: Clinical Relevance, Challenges, and Advances

**DOI:** 10.31083/RCM47007

**Published:** 2025-12-24

**Authors:** Kamran Namjouyan, Ervin Sejdić, Mark S. Link, Antonio Pelliccia, Benjamin Glicksberg, Natalia Trayanova, Chayakrit Krittanawong

**Affiliations:** ^1^Department of Cardiology, Geisinger Medical Center, Danville, PA 17821, USA; ^2^Edward S. Rogers Department of Electrical and Computer Engineering, University of Toronto, Toronto, ON M5S 3G4, Canada; ^3^North York General Hospital, Toronto, ON M2K 1E1, Canada; ^4^Department of Medicine, Division of Cardiology, University of Texas Southwestern Medical Center, Dallas, TX 75390, USA; ^5^Department of Medicine, Institute of Sports Medicine and Science, 00197 Rome, Italy; ^6^Hasso Plattner Institute for Digital Health, Icahn School of Medicine at Mount Sinai, New York, NY 10029, USA; ^7^Department of Biomedical Engineering, Johns Hopkins University, Baltimore, MD 21218, USA; ^8^Department of Medicine, Johns Hopkins University School of Medicine, Baltimore, MD 21205, USA; ^9^Alliance for Cardiovascular Diagnostic and Treatment Innovation (ADVANCE), Johns Hopkins University, Baltimore, MD 21218, USA; ^10^HumanX, Delaware, DE 19801, USA

**Keywords:** electrocardiogram, cardiovascular diagnostics, ECG interpretation, non-invasive cardiac assessment, arrhythmia detection, risk stratification, patient outcomes, sudden cardiac death, ECG screening

## Abstract

Electrocardiograms (ECGs) remain a foundational pillar of cardiovascular diagnostics, providing rapid, non-invasive diagnosis and being universally accessible to all clinicians. An ECG captures the electrical signals of the heart via a standard 12-lead configuration, offering insights into arrhythmias, conduction delays, ischemic injury, structural remodeling, and systemic pathologies with cardiac implications. This review presents a structured framework for ECG interpretation by discussing general approaches to rate, rhythm, axis, intervals, and repolarization dynamics, and by outlining both cardiac and non-cardiac conditions associated with ECG abnormalities. We explore the accelerating pace of innovations in artificial intelligence (AI) for ECG analysis. Deep learning algorithms now rival and, in select domains, surpass expert clinicians in detecting left ventricular systolic and diastolic dysfunction, hypertrophic obstructive cardiomyopathy, and acute myocardial infarction. The integration of AI-enhanced ECG interpretation enables earlier disease recognition, refined risk stratification, and optimized clinical decision-making across acute and chronic care settings. This review systematically guides readers through ECG interpretation, linking fundamental principles with nuanced clinical patterns using AI to enhance accurate diagnosis and improve patient outcomes across a wide range of cardiovascular conditions.

## 1. Introduction

An electrocardiogram (ECG) is a fast, noninvasive, and widely accessible diagnostic tool used to evaluate the electrical activity of the heart. By placing electrodes on the chest wall it captures and records the heart’s electrical signals, producing a visual trace that reflects the timing and strength of these impulses. This graphical output enables healthcare providers to assess several critical aspects of cardiac function such as heart rate, rhythm, regularity, the integrity of electrical conduction pathways, and signs of myocardial ischemia, infarction, or structural abnormalities such as hypertrophy or cardiomyopathies. The standard 12-lead ECG remains an important cardiac diagnostic due to its broad clinical utility. It is especially valuable in the initial evaluation of chest pain, palpitations, syncope, and other symptoms suggestive of heart disease. Its widespread use is supported by several key advantages: it is safe, painless, easy to perform, highly reproducible, and cost-effective [1, 2].

According to the American College of Cardiology (ACC) and the American Heart Association (AHA), ECG is considered the gold standard for noninvasive diagnosis of arrhythmias and conduction disturbances. These societies emphasize its role in both emergencies and routine cardiac care, and they emphasize its value not only in acute settings but also in long-term monitoring and risk stratification [1, 2]. Furthermore, updated practice standards underscore the importance of appropriate ECG use, including continuous monitoring in hospitalized patients, and ambulatory monitoring. Evidence-based protocols optimize its diagnostic yield. Overall, ECG is a central aspect of cardiovascular evaluation and can assist to guide clinical management in acute and long-term risk stratification in a variety of clinical situations and settings. We conducted a narrative review to guide readers through ECG interpretation by linking core principles with complex clinical patterns to improve cardiovascular diagnosis and patient outcomes. A formal literature search was performed using institutional databases such as PubMed, Scopus, Embase, and Web of Science, focusing on peer-reviewed articles published between 2015 and 2025. Keywords included “ECG interpretation”, “cardiovascular diagnosis”, “arrhythmia”, “atrial fibrillation”, “conduction disturbances”, “artificial intelligence”, “deep learning”, and “patient outcomes”. Relevant studies were selected based on their contribution to diagnostic frameworks and clinical applications, and thematically synthesized to highlight current practices, innovations, and gaps in ECG-based cardiovascular care.

## 2. Basic ECG Analysis

The systematic interpretation of an ECG is a crucial skill in clinical cardiology, involving a step-by-step approach to analyzing the heart’s electrical activity as recorded from the body’s surface. The process begins with the accurate placement of electrodes for a standard 12-lead ECG, which captures electrical signals from multiple anatomical angles. The recording is typically performed while the patient is at rest to ensure clear and reliable tracing. Before interpretation begins, it is essential to verify patient identification, confirm proper calibration settings, and check for any technical artifacts that could distort the results. Once the tracing is validated, the analysis proceeds in a structured manner. Clinicians assess the heart rate, rhythm (including its regularity and origin), and examine key waveform components such as the P wave morphology, PR interval, QRS complex duration and configuration, QT interval, and ST segment and T wave changes. Additional steps include determining the electrical axis of the heart and comparing the current ECG with previous tracings to detect new or evolving abnormalities. This comprehensive approach enables the identification of a wide range of cardiac conditions, including arrhythmia, conduction blocks, myocardial ischemia or infarction, chamber enlargement, and other structural or metabolic abnormalities.

### 2.1 Rate

Heart rate assessment on an ECG involves calculating the number of cardiac cycles per minute by analyzing the spacing between R waves, known as R-R intervals. On a standard 12-lead ECG recorded at a paper speed of 25 mm/sec, two commonly used methods can be used. The first is the 300 method, ideal for regular rhythms: by counting the number of large (5 mm) boxes between two consecutive R waves and dividing 300 by that number, clinicians can estimate the heart rate in beats per minute (bpm). The second is the 10-second method, which is particularly useful for irregular rhythms such as atrial fibrillation. This approach involves counting the number of R waves in a 10-second strip and multiplying by 6 to approximate bpm. For greater accuracy in irregular rhythms, longer recordings, such as 30 or 60 seconds are recommended [3].

### 2.2 Rhythm

Cardiac rhythm analysis on an ECG involves evaluating the pattern and regularity of the heart’s electrical impulses, which coordinate its contractions. This process begins by determining whether the rhythm originates from the sinus node of the heart’s natural pacemaker and whether the intervals between beats are consistent. Clinicians assess the P waves, which represent atrial depolarization, their relationship to the QRS complexes (ventricular depolarization), and the regularity of R-R intervals. A normal sinus rhythm is identified by upright P waves in lead I and II that precede each QRS complex at a steady rate. Deviations from this pattern suggest arrhythmias, which may be supraventricular (e.g., atrial fibrillation, atrial flutter) or ventricular (e.g., ventricular tachycardia, ventricular fibrillation) in origin. According to the ACC/AHA, the ECG remains the gold standard for noninvasive diagnosis of arrhythmias and conduction disturbances [1]. Accurate rhythm interpretation is essential for identifying conditions such as bradyarrhythmia, tachyarrhythmia, and conduction blocks, and is a cornerstone of effective cardiac care.

### 2.3 Axis

The electrical axis on an ECG represents the average direction of ventricular depolarization within the frontal plane, evaluated using the QRS complex. It is assessed by examining the overall positive or negative deflections of the QRS complexes in limb leads, especially leads I and aVF or through more detailed vector analysis using the hexaxial reference system. According to guidelines from the AHA/ACC/Heart Rhythm Society (HRS), a normal adult QRS axis ranges from –30° to 90°. Left-axis deviation is defined as an axis between –30° and –90°, while right-axis deviation falls between 90° and 180°. If the QRS complexes appear equiphasic in the limb leads, the axis is considered indeterminate [4]. In pediatric populations, the normal axis tends to be more rightward and gradually shifts leftward with age, as outlined in clinical guidelines. The electrical axis is a key component of basic ECG interpretation, offering valuable diagnostic insights into potential cardiac or systemic conditions. Deviations in axis can suggest conduction abnormalities (such as fascicular blocks), chamber enlargement, or the presence of an old or new MI [4].

### 2.4 Intervals

The PR interval represents the time between the onset of atrial depolarization and the beginning of ventricular depolarization. It is measured from the start of the P wave to the start of the QRS complex. According to the AHA, ACC, and HRS, a normal PR interval in adults ranges from 120 to 200 milliseconds. A PR interval longer than 200 ms suggests first-degree atrioventricular (AV) block, while a shorter interval, less than 120 ms may indicate pre-excitation syndromes (such as Wolff-Parkinson-White) or junctional rhythms. Although a prolonged PR interval is often benign, it can be a marker of underlying conduction system disease in certain clinical contexts [4].

The QRS complex duration reflects the time required for ventricular depolarization. A normal QRS duration is ≤120 milliseconds in adults. A duration exceeding this threshold may point to bundle branch blocks or intraventricular conduction delays. While QRS duration can vary based on age, sex, and measurement technique, a value greater than 120 ms is typically considered indicative of a significant conduction abnormality [4].

The QT interval spans from the beginning of ventricular depolarization to the end of repolarization and measured from the start of the QRS complex to the end of the T wave. Because the QT interval varies with heart rate, it is commonly corrected (QTc) using formulas such as Bazett’s. The AHA/ACC/HRS define a prolonged QTc as ≥450 ms in men and ≥460 ms in women (with 99th percentile being 470 ms in males and 480 ms in females), while a QTc ≤330 ms is considered abnormally short [4]. A prolonged QTc is associated with an increased risk of torsades de pointes and sudden cardiac death, whereas a short QTc may also predispose to arrhythmias [5].

During routine ECG interpretation, these intervals are systematically measured and compared to established normal values. Abnormalities may signal conduction system disease, electrolyte imbalances, drug effects, or inherited channelopathies, and should prompt further clinical evaluation when appropriate.

### 2.5 ST Segment and T-Wave

The ST segment and T wave are vital components of the ECG for evaluating ventricular repolarization and plays a role in identifying conditions such as myocardial ischemia or infarction, pericarditis, electrolyte imbalances, and drug effects. The AHA/ACC/HRS recommend assessing the ST segment for elevation or depression relative to the TP or PR segment, with measurements taken at the J point and again 40–80 milliseconds afterward in certain clinical scenarios. ST-segment elevation may indicate acute myocardial injury, pericarditis, or early repolarization. Abnormal thresholds for J-point elevation in leads V2–V3 are ≥0.2 mV in men ≥40 years, ≥0.25 mV in men <40 years, and ≥0.15 mV in women; in all other leads, ≥0.1 mV is considered abnormal. The morphology (upsloping, horizontal, or downsloping) and distribution of ST changes are essential for distinguishing ischemic from non-ischemic causes [5].

**Fig. 1.**
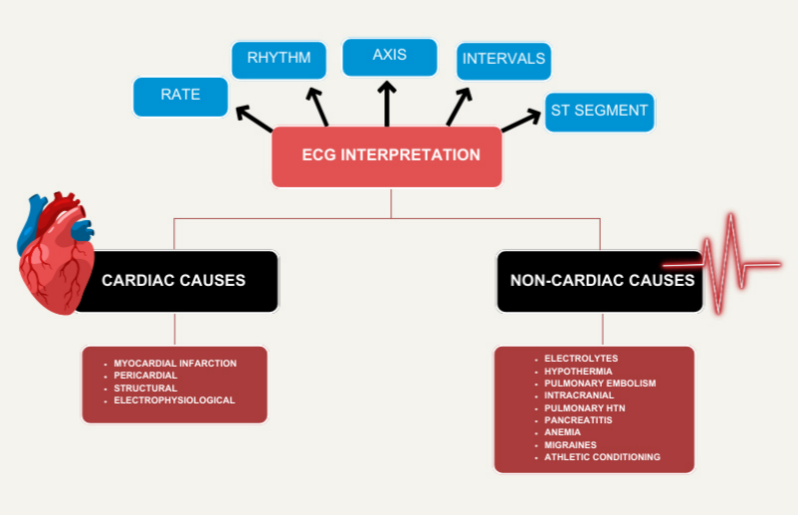
**General approach to ECG interpretation**. ECG, electrocardiogram. The figure was created with https://www.canva.com.

ST-segment depression is most associated with subendocardial ischemia, though it may also result from electrolyte disturbances, medications, or conduction abnormalities. Similarly, T-wave abnormalities including inversion, flattening, or hyperacute changes can reflect primary repolarization disturbances such as ischemia, myocarditis, or electrolyte imbalance, or secondary changes due to altered depolarization patterns, such as those seen with bundle branch blocks or ventricular pacing. Careful interpretation of these repolarization features is essential for accurate diagnosis and clinical decision-making. Fig. 1 illustrates a general clinician approach to ECG interpretation.

## 3. Common ECGs in Clinical Practice

### 3.1 Cardiac Etiology

#### 3.1.1 Myocardial Infarction

MI suspicion is one of the main reasons many clinicians acquire an ECG as this is life threatening and timely clinical intervention is essential. According to the AHA, ST-segment elevation myocardial infarction (STEMI) is typically identified by new ST elevation at the J point in at least two contiguous leads. This is often accompanied by reciprocal ST depression in opposing leads. The presence of new Q waves suggests myocardial necrosis, while T wave inversions or hyperacute T waves may serve as early indicators of ischemia. In contrast, non–ST-elevation myocardial infarction (NSTEMI) usually presents with ST depression and/or T wave inversion, without ST elevation. Additionally, a new left bundle branch block (LBBB), when seen in the appropriate clinical context, may also signify an acute MI [6].

Occlusion of the left main coronary artery (LMCA) typically results in extensive and severe myocardial ischemia. On ECG, this often appears as widespread ST-segment depression (≥1 mm in six or more leads), most prominently in the lateral and inferior leads, accompanied by ST-segment elevation in lead aVR and sometimes V1. This pattern reflects significant ischemia and is associated with a high risk of cardiogenic shock and poor clinical outcomes. The AHA highlights that this ECG presentation is highly suggestive of critical LMCA or severe multivessel coronary artery disease, particularly when accompanied by hemodynamic instability. In rare and extreme cases, a distinctive “triangular” or “lambda-like” QRS-ST-T waveform may be observed, indicating profound ischemia or impending cardiac collapse [6, 7, 8].

Proximal occlusion of the left anterior descending (LAD) artery typically produces extensive ECG changes, with ST-segment elevation across the anterior precordial leads (V1–V6), lead I, and aVL, often accompanied by reciprocal ST depression in the inferior leads. This widespread pattern reflects the large myocardial territory at risk, including the anterior wall, septum, and frequently part of the lateral wall. In contrast, occlusion of a diagonal branch of the LAD generally results in a more localized pattern, most commonly ST-segment elevation in leads V2–V3, sometimes extending into lead aVL, with reciprocal ST depression in leads II and III. This more focal distribution corresponds to anterolateral wall ischemia. Recognizing these distinct ECG signatures helps differentiate between a major proximal LAD event and an isolated diagonal branch lesion, which will support the diagnosis of an acute anterior myocardial infarction in the LAD territory [9]. Left circumflex artery (LCx) occlusion is frequently underdiagnosed on standard 12-lead ECG due to its subtle or atypical presentation. The AHA notes that horizontal or downsloping ST-segment depression in leads V1 to V3 especially when accompanied by ST elevation in posterior leads V7 to V9 suggests posterior myocardial infarction, often due to LCx occlusion. Additionally, LCx involvement may produce ST elevation in lateral leads (I, aVL, V5, V6), and if the LCx is the dominant vessel, it can also cause ST elevation in inferior leads (II, III, aVF). Because posterior infarction may not be evident on standard leads, posterior leads are essential for accurate diagnosis, as isolated precordial ST depression may be the only clue [6].

Right coronary artery (RCA) occlusion typically presents with ST-segment elevation in the inferior leads II, III, and aVF with lead III showing greater elevation than lead II. Reciprocal ST-segment depression is often seen in leads I and aVL. When the occlusion is proximal, it may also involve the right ventricle, which can be detected by ST-segment elevation in right-sided precordial leads such as V3R and V4R, and occasionally in V1. The ACC and AHA emphasize the importance of obtaining a right-sided ECG in suspected cases of right ventricular infarction, as this can significantly improve diagnostic accuracy and guide appropriate management, especially in hemodynamically unstable patients [9].

In addition to the above features, many other ECG patterns have been correlated with acute MI and are essential in timely diagnosis and should not be missed in clinical settings. Several nontraditional ECG patterns can signal acute coronary occlusion and warrant urgent reperfusion therapy, even in the absence of classic ST-segment elevation. Posterior MI often presents as horizontal ST depression in leads V1–V3, accompanied by tall R waves and upright T waves; ST elevation in posterior leads V7–V9 confirms transmural posterior infarction, a STEMI equivalent. Wellens syndrome, marked by deeply inverted or biphasic T waves in V2–V3 during pain-free intervals, indicates critical proximal LAD stenosis and a high risk of anterior MI 2. Hyperacute T waves broad, symmetric, and prominent may precede ST elevation and are an early sign of coronary occlusion [6, 10].

Additional high-risk patterns include the de Winter sign, characterized by upsloping ST depression at the J-point in V1–V4 with tall, symmetric T waves, typically seen in proximal LAD occlusion. New or presumed new left bundle branch block (LBBB) can obscure STEMI; the modified Sgarbossa criteria improve diagnostic accuracy in this setting. Terminal QRS distortion, defined by the absence of both the S wave and J wave in V2–V3, is a specific marker of anterior STEMI and helps differentiate it from early repolarization. Moreover, the combination of new right bundle branch block with left anterior fascicular block, especially in the presence of ischemic symptoms, suggests extensive anterior infarction. Prompt recognition of these patterns is critical, as they are associated with high morbidity and mortality and require immediate reperfusion therapy [6, 10, 11, 12].

#### 3.1.2 Pericardial Involvement

There are various ECG patterns that can help clinicians in detecting pericardial involvement. Acute pericarditis typically presents with distinctive ECG findings, most notably widespread concave (upward) ST-segment elevation and PR-segment depression. These abnormalities are generally diffuse, affecting the majority of limb and precordial leads particularly leads I, II, III, aVL, aVF, and V2–V6 while sparing aVR and often V1. The PR-segment depression is especially prominent in the inferior and precordial leads. These changes are most evident during the early phase of the illness. However, these ECG features are not universally observed. Only about 25–50% of patients with acute pericarditis exhibit these classic changes, and the ECG may appear normal in cases that are mild or promptly treated [13, 14].

Aside from pericarditis, ECG patterns can be beneficial in detecting effusions which can be a complication of it. Pericardial effusion refers to the accumulation of fluid within the pericardial sac, which may be serous, hemorrhagic, or purulent in nature. Effusions are typically categorized by volume small (50–100 mL), moderate (100–500 mL), and large (>500 mL) as well as by their temporal profile (acute vs. chronic). Importantly, the clinical impact is more closely related to the rate of fluid accumulation than to the absolute volume. Rapid accumulation, even of modest amounts, can precipitate cardiac tamponade, whereas slow accumulation may be tolerated even at larger volumes [15]. The primary clinical concern with pericardial effusion is the potential for hemodynamic compromise. Cardiac tamponade represents the most severe complication, characterized by impaired ventricular filling, hypotension, elevated jugular venous pressure, and pulsus paradoxus which is a drop in systolic blood pressure >10 mmHg during inspiration [15, 16]. Symptoms of pericardial effusion are often vague, including dyspnea and chest discomfort, and physical examination findings may be unreliable. Although echocardiography is the gold standard of detecting effusions, ECG can be useful in some cases. Although it is routinely included in the initial evaluation of patients with suspected pericardial disease, its findings lack both sensitivity and specificity for detecting effusions. The most recognizable ECG features associated with large pericardial effusions are low QRS voltage and electrical alternans, a beat-to-beat variation in QRS amplitude and axis caused by the heart swinging within the fluid-filled pericardial sac. These findings are more commonly seen in cases of large effusions or when cardiac tamponade is present. However, they are not consistently observed and may be absent in smaller or slow accumulating effusions. As such, ECG should not be relied upon for definitive diagnosis, and echocardiography remains the gold standard for evaluating pericardial effusion and its hemodynamic impact [13].

#### 3.1.3 Structural Abnormalities

Hypertrophic cardiomyopathy (HCM) is a genetic disorder of the sarcomere, marked by asymmetric thickening of the left ventricular wall, most commonly involving the interventricular septum. This hypertrophy can lead to dynamic obstruction of the LV outflow tract, either at rest or during physiological provocation. It is described as myocyte disarray, interstitial fibrosis, and impaired diastolic filling. These structural and functional abnormalities contribute to an increased risk of arrhythmia [17]. A standard 12-lead ECG reveals abnormalities in approximately 75–95% of individuals with phenotypic HCM, including features such as increased voltage suggestive of left ventricular hypertrophy, repolarization abnormalities, pathologic Q waves, and pseudo-infarct patterns. However, ECG findings do not consistently correlate with the extent or anatomical distribution of hypertrophy. According to the ACC/AHA, a 12-lead ECG should be performed during the initial evaluation and repeated every 1–2 years. It is also recommended as part of routine screening for first-degree relatives. While a normal ECG reduces the likelihood of significant hypertrophy detectable by cardiac MRI, it does not definitively exclude it [18, 19]. Certain ECG features such as pseudo-ST elevation, prolonged QRS duration, low voltage, and extended QTc are independently linked to worse outcomes in HCM. These markers can complement imaging and clinical data to improve risk stratification [20].

Arrhythmogenic Right Ventricular Cardiomyopathy (ARVC) is a genetic heart muscle disorder marked by progressive fibrofatty replacement of the right ventricular myocardium, especially the free wall. This structural remodeling predisposes patients to ventricular arrhythmias, RV dysfunction, and sudden cardiac death (SCD). ECG plays a role in both diagnosing ARVC and assessing arrhythmic risk. Patients with ARVC have T-wave inversions in the anterior leads. One of the hallmark ECG findings is the epsilon wave, which is a low-amplitude signal following the QRS complex in right precordial leads indicating delayed right ventricular activation due to fibrofatty scarring, usually present in the advanced stage of the disease. However, the majority of patients with ARVC will not have this finding. Signal-averaged ECG (SAECG) can detect late potentials, which serve as a minor criterion when the standard QRS duration is ≤110 ms. These include a filtered QRS duration ≥114 ms, terminal signal <40 µV lasting >37 ms, or a root-mean-square voltage in the terminal 40 ms ≤20 µV. Abnormal SAECG findings have been linked to more severe disease on cardiac MRI and a higher incidence of adverse outcomes, mainly in males [21].

### 3.2 Non-Cardiac Etiologies

#### 3.2.1 Electrolytes

Electrolyte imbalances, particularly potassium, calcium, and magnesium can lead to distinctive ECG changes and potentially life-threatening arrhythmias. Hypokalemia typically presents with ECG findings such as T-wave flattening, ST-segment depression, prominent U waves, and QTc prolongation. These abnormalities get stronger as serum potassium levels drop, increasing the risk of arrhythmias like atrial fibrillation, ventricular tachycardia, torsades de pointes, and ventricular fibrillation. According to the AHA, classic ECG indicators include broad T waves, ST depression, and prominent U waves increase the risk of arrythmia when potassium falls below 3.0 mEq/L [22]. In contrast, hyperkalemia produces a progressive series of ECG changes as potassium levels rise. Initially, peaked T waves appear at levels between 5.5 and 6.5 mmol/L, followed by PR interval prolongation (6.5–7.5 mmol/L), QRS widening (7.0–8.0 mmol/L), and, in extreme cases, a sine wave pattern, ventricular fibrillation, or asystole when levels exceed 10 mmol/L. The AHA cautions that these changes may vary between individuals and are not always predictable, thus clinical judgement should be used for all cases [22, 23].

Hypocalcemia is associated with QT interval prolongation, primarily due to ST segment lengthening, which heightens the risk of torsades de pointes. Conversely, hypercalcemia shortens the QT interval by abbreviating the ST segment. Hypomagnesemia can also lead to QT prolongation and predispose patients to torsades de pointes, often in conjunction with hypokalemia. On the other hand, hypermagnesemia may cause PR and QRS prolongation, and at very high concentrations, it can result in complete heart block or asystole [24].

#### 3.2.2 Hypothermia

Hypothermia induces distinct and progressively worsening ECG changes that closely correlate with the degree of core temperature reduction. The hallmark finding is the Osborn (J) wave, which is a positive deflection at the junction of the QRS complex and the ST segment which becomes increasingly prominent as hypothermia deepens. Additional ECG abnormalities that can occur in up to half of patients include sinus bradycardia, prolongation of the PR, QRS, and QT intervals, and atrial fibrillation. As core temperatures fall below 28 °C, the risk of life-threatening ventricular arrhythmias, such as ventricular fibrillation rises significantly. These changes are observed in both accidental and therapeutic hypothermia, with their severity and frequency intensifying at lower temperatures. According to the AHA, hypothermia slows cardiac conduction, leading to interval prolongation and the appearance of Osborn waves that their amplitude reflects the degree of hypothermia. Other possible findings include ST-segment elevation or depression, T-wave abnormalities, and, in rare cases, ECG patterns resembling Brugada syndrome or pericarditis, potentially complicating the diagnosis of acute ischemia or other cardiac conditions in hypothermic patients [22, 25].

#### 3.2.3 Pulmonary Embolism (PE) 

PE is associated with several ECG findings, most commonly sinus tachycardia, T-wave inversion in the right precordial leads (V1–V4), the classic S1Q3T3 pattern (characterized by an S wave in lead I, a Q wave in lead III, and T-wave inversion in lead III), and right bundle branch block. These changes reflect acute right ventricular strain and are more frequently observed in cases of massive or submassive PE. Additional ECG abnormalities may include right axis deviation, ST-segment elevation in lead aVR, and atrial arrhythmias such as atrial fibrillation. However, it is important to recognize that up to 20–25% of patients with PE may present with a normal ECG, and none of these findings are sufficiently sensitive or specific to confirm the diagnosis and clinical judgment should be used in all cases [26, 27]. According to joint guidelines from the ACC, ECG is generally insensitive for detecting PE but may reveal signs of right heart strain, with T-wave inversion in V1–V4 offering the greatest accuracy for identifying right ventricular dysfunction. Recent studies and meta-analyses support that sinus tachycardia is the most frequent ECG abnormality, followed by T-wave inversion in V1–V3, S1Q3T3, and right bundle branch block, though the overall diagnostic utility of these findings remains limited [28].

#### 3.2.4 Intracranial Pathologies

ECG changes associated with intracranial pathologies most commonly include QT interval prolongation, T-wave inversion, ST-segment depression, and prominent U waves. Less frequently, findings such as ST-segment elevation, sinus bradycardia, sinus tachycardia, and various arrhythmias including atrial fibrillation and ventricular ectopy may also be observed. These ECG abnormalities are particularly prevalent in acute cerebrovascular events, such as subarachnoid hemorrhage, intracerebral hemorrhage, and large ischemic strokes, and can closely mimic patterns seen in ACS, potentially complicating the diagnostic process [29].

QTc prolongation is the most frequently observed ECG abnormality in intracerebral hemorrhage. It is linked to insular cortex involvement, larger hematoma volumes, and intraventricular extension. T-wave inversion is also common and has been independently associated with increased mortality in these patients [30]. Other ECG changes include ST-segment depression and less commonly, ST-segment elevation maybe present with J wave-like morphology. Prominent U waves and conduction abnormalities, such as bundle branch blocks have also been reported. Arrhythmias including bradycardia, supraventricular tachycardias, and ventricular arrhythmias can further complicate the clinical course, particularly in cases involving the right insular cortex due to autonomic dysregulation. These ECG changes typically evolve over several days and may resolve within two weeks but in some instances such as QT prolongation and U waves, can persist beyond that period [31, 32].

#### 3.2.5 Pulmonary Hypertension

Pulmonary hypertension is commonly associated with ECG findings that reflect chronic pressure overload and structural remodeling of the right heart. The most frequent abnormalities include right axis deviation, right ventricular hypertrophy, and right ventricular strain patterns. A review published in JAMA reported that right axis deviation occurs in approximately 79% of cases, right ventricular hypertrophy in 87%, and right ventricular strain in 74% [33]. These changes are indicative of elevated pulmonary artery pressures and their impact on cardiac function. Additional ECG features often seen in pulmonary hypertension include right atrial enlargement (P pulmonale), right bundle branch block, deep R waves in leads V1 and V2, deep S waves in leads V5 and V6, and repolarization abnormalities such as ST-segment depression or T-wave inversion in the inferior and right precordial leads (II, III, aVF, V1–V3). Composite patterns such as the combination of right axis deviation, right ventricular hypertrophy, and right atrial enlargement are associated with more severe hemodynamic compromise and poorer clinical outcomes [34, 35].

#### 3.2.6 Pancreatitis

In the context of acute pancreatitis, ECG abnormalities often resemble those seen in ACS. The most frequently observed changes include nonspecific repolarization abnormalities such as T-wave flattening or inversion and ST-segment depression along with sinus tachycardia and QTc prolongation. Although less common, ST-segment elevation, particularly in the inferior leads, may occur and can closely mimic acute MI even in the absence of underlying coronary artery disease. QTc prolongation is noted in over half of patients with acute pancreatitis and has been linked to increased mortality, especially when accompanied by diastolic dysfunction or pericardial effusion. In severe cases, both QTc dispersion and maximum QTc interval are elevated and correlate with clinical severity scores, such as the Ranson criteria [36, 37]. Additional ECG findings reported in acute pancreatitis include left anterior hemiblock, an abnormal QRS-T angle, and less commonly pericardial effusion. Notably, the QRS-T angle may serve as a useful marker in distinguishing between mild and severe forms of the disease [38].

#### 3.2.7 Anemia

ECG changes in patients with anemia are generally nonspecific but it can include several abnormalities. The most consistently reported findings are QT and QTc interval prolongation, reduced T-wave amplitude, and decreased QRS complex voltage. These changes tend to become more pronounced with increasing severity of anemia and are thought to result from both diminished oxygen delivery to the myocardium and altered blood resistivity. This phenomenon is known as the Brody effect [39]. In cases of iron deficiency anemia, additional ECG alterations have been observed. These include increased P-wave duration and dispersion, prolonged QT and QTc intervals and dispersion, and elevated Tp interval. These changes may reflect heightened susceptibility to atrial and ventricular arrhythmias, underscoring the potential cardiac risks associated with severe or untreated iron deficiency [40].

#### 3.2.8 Migraines

ECG changes associated with migraines are most commonly observed during acute attacks and are thought to reflect autonomic dysregulation. The most consistently reported abnormalities include prolonged QT and QTc intervals, increased QTc dispersion, and elevated P-wave dispersion changes that tend to normalize between episodes and are significantly more pronounced during migraine attacks compared to pain-free periods and healthy controls [41]. During migraine episodes, studies have also noted a higher frequency of T-wave inversion, ST-segment abnormalities, and less commonly rhythm disturbances such as sinus arrhythmia, atrial premature contractions, and ventricular premature contractions. PR interval prolongation has been reported as well, particularly in patients with frequent attacks and in male migraine sufferers [42].

## 4. Artificial Intelligence (AI) Interpretation of the Electrocardiogram

### 4.1 AI-ECG in LV Dysfunction

**Fig. 2.**
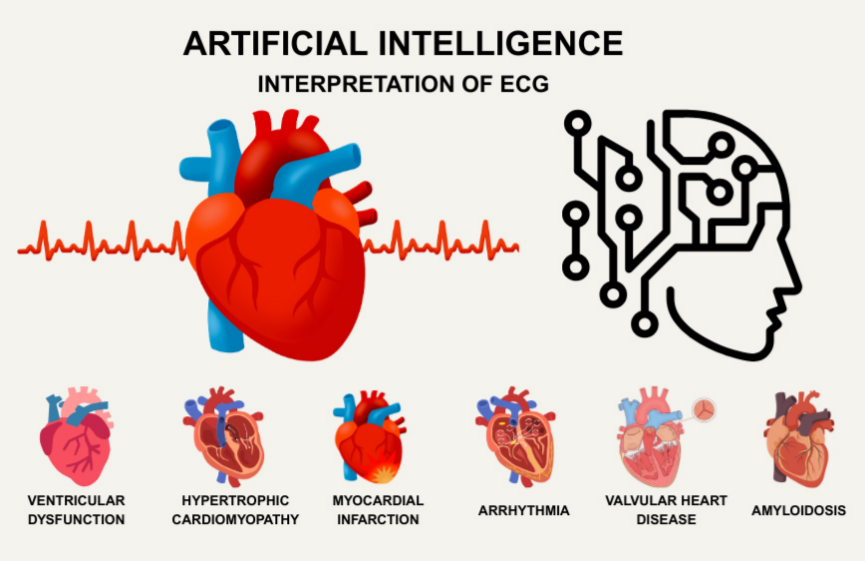
**Overview of AI-ECG performance across multiple cardiology domains, highlighting its strengths in diagnosis and risk assessment**. The figure was created with https://www.canva.com.

Recent advancements in AI have significantly enhanced ECG analysis. It has brought improvement in both diagnostic precision and the efficiency of clinical workflows. Deep learning techniques, particularly convolutional neural networks now support automated ECG interpretation with performance comparable to or in some cases exceeding that of expert clinicians across various cardiovascular conditions, such as arrhythmia, left ventricular dysfunction (LVD), HCM, and acute MI [43]. In its 2024 scientific statement, the AHA emphasizes the potential of AI-enhanced ECG analysis to detect hidden structural heart diseases such as LVD up to one to two years earlier than conventional diagnostic approaches. Additionally, AI algorithms have demonstrated high accuracy in identifying conditions like HCM, amyloid heart disease, and pulmonary hypertension. Prospective studies further suggest that AI-ECG tools can enhance the detection of ventricular dysfunction and asymptomatic atrial fibrillation, while also showing promise in stratifying risk for major adverse cardiovascular events [44]. Table 1 (Ref. [45, 46, 47, 48, 49, 50, 51, 52, 53, 54, 55, 56]) provides a comprehensive summary of notable recent studies that have applied AI to interpret ECGs for cardiovascular diagnostics. Each entry outlines the study’s author and year, design type, data source, primary aim, and key findings or comments. The studies span a wide range of cardiovascular conditions including heart failure, cardiac amyloidosis (CA), arrhythmias, valvular diseases, myocardial infarction, and atherosclerotic cardiovascular disease. It demonstrated that how AI-enhanced ECG models can improve diagnostic accuracy and even clinical outcomes. The table highlights the scalability, sensitivity, and specificity of AI algorithms by showing their potential to outperform traditional methods and support personalized, timely interventions in both inpatient and outpatient settings. Furthermore, Fig. 2 offers a comprehensive overview of AI-ECG’s capabilities across different areas of cardiology.

**Table 1.** **Summary of remarkable AI interpreted ECG studies**.

Author, year	Study type/Design	Data collection	Study aim	Author comments
Attia *et al*., 2019 [49]	Retrospective	>50k ECGs in an independent test set from Mayo Clinic health system.	Detect low LVEF (≤35–40%) from standard 12-lead ECG.	The AI-enabled ECG model achieved an AUC of 0.93 with 86.3% sensitivity, 85.7% specificity and accuracy, and identified patients without ventricular dysfunction but with a positive screen as having a fourfold increased risk of future dysfunction (HR 4.1; 95% CI: 3.3–5.0).
				AI-ECG accurately identified low EF from routine ECGs, supporting its use as a non-invasive screening tool.
Dhingra *et al*., 2025 [50]	Retrospective	n = 192,667 patients with baseline ECGs.	To evaluate the ability of AI model to predict the risk of heart failure using single-lead ECGs and compare its performance to traditional clinical risk factors.	The AI-ECG model effectively forecasts the risk of future heart failure and demonstrates superior performance compared with conventional risk assessment methods. Combining AI-ECG predictions with standard clinical risk factors further improves accuracy, offering the potential to guide personalized interventions and optimize patient outcomes.
Grogan *et al*., 2021 [48]	Retrospective	n = 2541 patients with light chain or transthyretin CA seen at Mayo Clinic between 2000–2019.	To detect CA from 12-lead ECG via AI.	The AI-enhanced ECG model achieved an AUC of 0.91 and detected 84% of holdout CA cases.
				It predicted cardiac amyloid over six months before clinical diagnosis in 59% of patients with prior ECGs.
Kalmady *et al*., 2024 [51]	Retrospective	1,605,268 ECGs, 244,077 adults, 84 hospitals.	Develop/validate AI-ECG for 15 CV diagnoses at population level.	Learning models achieved AUROCs >90% for four cardiovascular conditions and outperformed XGB models by ∼5%, demonstrating good-to-excellent diagnostic accuracy across 15 diagnoses. AI-enabled ECG models offer scalable, high-fidelity diagnostic support for diverse cardiovascular conditions, advancing population-level screening and early detection.
Lai *et al*., 2023 [52]	Retrospective	n = 658,486 wearable 12-lead ECGs.	To develop a robust, real-time diagnostic algorithm for wearable 12-lead ECGs using self-supervised learning on a large-scale dataset to detect 60 cardiac conditions.	The model achieved an AUROC of 0.975 and F1 score of 0.575 offline, and maintained strong performance online with 0.736 sensitivity and 0.954 specificity, demonstrating its potential for early, scalable arrhythmia detection.
Liang *et al*., 2025 [47]	Retrospective	n = 400,882 patients with 988,618 paired ECG and Echocardiogram.	To develop and validate AI-ECG models capable of diagnosing and predicting future moderate or severe regurgitant valvular heart diseases, including MR, TR, and AR.	AI-ECG models can accurately diagnose and predict future significant regurgitant valvular heart disease (MR, TR, AR) across diverse populations. These models hold potential to guide targeted echocardiographic surveillance, enabling earlier detection and intervention for patients at high risk of clinically significant valvular disease.
Lin *et al*., 2024 [53]	Multicenter, Randomized Clinic Trial	n = 15,965 hospitalized patients, 39 physicians, intervention vs. control.	To test if AI-ECG alerts reduce all-cause mortality.	Implementation of the AI-ECG alert reduced 90-day all-cause mortality from 4.3% to 3.6% (HR 0.83; 95% CI: 0.70–0.99), with greater benefit in high-risk ECG patients (HR 0.69; 95% CI: 0.53–0.90). AI-ECG alerts enable early identification of high-risk patients, prompting timely care and significantly improving survival.
Lee *et al*., 2025 [46]	Multicenter, Prospective Cohort	n = 8493 adults, 18 hospitals, ED patients with suspected acute MI.	To evaluate AI-ECG for detecting acute MI in ED.	AI-ECG achieved an AUROC of 0.878 for AMI and 0.866 for 30-day MACE, outperforming traditional scores with a net reclassification improvement of 19.6%. AI-enhanced ECG interpretation offers diagnostic and prognostic accuracy comparable to expert clinicians, supporting its integration into emergency cardiovascular care.
Raghunath *et al*., 2021 [54]	Retrospective	n = 1.6 million 12-lead ECG collected from 1984 to 2019.	To evaluate whether a deep neural network can predict new-onset AF from resting 12-lead ECGs in patients without prior AF and assess its utility in identifying individuals at elevated risk for AF-related stroke.	A deep neural network predicted new-onset AF within 1 year of a resting 12-lead ECG with an AUC of 0.85 and precision-recall AUC of 0.22. In a 30-year survival analysis, the hazard ratio for AF in high- vs. low-risk groups was 7.2 (95% CI: 6.9–7.6). Simulated deployment yielded 69% sensitivity, 81% specificity, and a number needed to screen of 9. Notably, 62% of patients who experienced AF-related stroke within 3 years had been classified as high risk. Deep learning applied to ECGs can effectively identify patients at elevated risk for future AF and AF-related stroke.
Yao *et al*., 2021 [45]	Pragmatic cluster-randomized trial	n = 22,641 adults, 120 primary care teams, intervention vs. control.	To assess if AI-ECG enables early diagnosis of low ejection fraction.	The intervention significantly increased new diagnoses of low ejection fraction (2.1% vs. 1.6%; OR 1.32, 95% CI: 1.01–1.61, *p* = 0.007), with even greater impact among AI-ECG–positive patients (19.5% vs. 14.5%; OR 1.43, 95% CI: 1.08–1.91, *p* = 0.01). AI-enabled ECG screening in routine care improves early detection of low ejection fraction, especially among high-risk individuals.
Zhang *et al*., 2024 [55]	Retrospective	n = 189,539 patients with 1,163,401 ECGs from a U.S. secondary care population.	To evaluate whether an AI-ECG model can improve prediction of 10-year ASCVD risk compared with the traditional ACC/AHA pooled cohort equations and established ASCVD risk factors.	AI-ECG models provide accurate prediction of 10-year ASCVD risk and demonstrate superior performance compared with both the pooled cohort equations and other AI-based ECG risk estimators. Incorporating AI-ECG with conventional risk factors further enhances predictive accuracy. These results highlight the potential of AI-ECG to optimize risk stratification, helping to prevent unnecessary treatment in low-risk individuals while ensuring timely preventive interventions for those at higher risk.
Zhao *et al*., 2020 [56]	Retrospective algorithm development	n = 667 STEMI ECGs, 7571 controls.	To develop AI for early STEMI detection.	The AI algorithm for STEMI detection achieved an AUC of 0.9954 (95% CI: 0.9885–1), with 96.75% sensitivity and 99.20% specificity, outperforming cardiologists whose sensitivity was 71.73% and accuracy 80.53%.
				AI-based ECG interpretation enables highly accurate, real-time STEMI diagnosis, surpassing expert clinicians and supporting faster, life-saving interventions.

ECG, Electrocardiogram; AI-ECG, AI-enhanced electrocardiogram; MI, myocardial infarction; STEMI, ST-elevation myocardial infarction; ACC, American College of Cardiology; AHA, American Heart Association; AUC, area under the curve; AF, atrial fibrillation; AR, aortic regurgitation; MR, mitral regurgitation; TR, tricuspid regurgitation; CA, cardiac amyloidosis; AMI, acute myocardial infarction; OR, odds ratio; ED, emergency department; LVEF, left ventricular ejection fraction; EF, ejection fraction; XGB, extreme gradient boosting; HR, hazard ratio; CI, confidence interval; AUROCs, area under the receiver operating characteristic curve; CV, cardiovascular; ASCVD, atherosclerotic cardiovascular disease; MACE, major adverse cardiovascular events.

AI-enhanced electrocardiogram (AI-ECG) has emerged as a valuable tool in the early detection and management of LVD, particularly left ventricular systolic dysfunction. By utilizing deep learning applied to standard 12-lead ECGs or even ECG images, AI-ECG enables non-invasive, scalable screening with high diagnostic accuracy typically achieving area under the curve (AUC) values between 0.90 and 0.97. It presents a strong sensitivity and specificity across a range of populations and clinical settings, including primary care, community-based screening, and critical care environments [45, 57]. Prospective randomized trials have shown that integrating AI-ECG clinical decision support into routine care significantly improves the diagnosis of low ejection fraction (≤50%) by prompting timely and appropriate echocardiographic evaluation without increasing unnecessary imaging [58].

### 4.2 AI-ECG in Diastolic Function

Diastolic dysfunction is characterized by impaired relaxation and/or increased stiffness of the left ventricle, leads to abnormal ventricular filling and elevated filling pressures. It is a central pathophysiological mechanism in heart failure with preserved ejection fraction. While echocardiographic assessment remains the standard for diagnosis, its utility may be limited in certain clinical settings due to indeterminate findings or lack of access. AI-ECG analysis has emerged as a promising noninvasive approach for detecting diastolic dysfunction. Deep learning models trained on large datasets of paired ECG and echocardiographic data can identify subtle ECG features indicative of diastolic dysfunction and elevated filling pressures features that may not be readily apparent to human interpreters. Recent studies have demonstrated that AI-ECG models can accurately predict echocardiographically defined grades of diastolic dysfunction and elevated filling pressures, with AUC values exceeding 0.9 for more advanced dysfunction and pressure elevations [59].

### 4.3 AI-ECG in Hypertrophic Obstructive Cardiomyopathy

AI-ECG is increasingly recognized for its potential in detecting HOCM, a subtype of HCM characterized by left ventricular outflow tract obstruction. Deep learning models, particularly those utilizing convolutional neural networks have demonstrated consistently high diagnostic performance for HCM with AUC values exceeding 0.90. Reported sensitivity ranges from 67% to 92%, and specificity from 88% to 99%, across varied populations and clinical environments based on the literature [60, 61]. Implementation studies have demonstrated that AI-ECG can be effectively integrated into routine clinical workflows to identify individuals at risk for HOCM prompting timely follow-up and diagnostic evaluation. This approach facilitates earlier detection and helps address under-recognition of HCM, particularly in underserved populations given low access to healthcare [62]. Additionally, AI-ECG models have demonstrated the ability to detect high-risk imaging features associated with adverse clinical outcomes such as severe hypertrophy and apical aneurysms. This capability may support more targeted use of advanced imaging modalities and enhance risk stratification strategies [63].

### 4.4 AI-ECG in AMI

AI-ECG has shown strong diagnostic performance in identifying acute MI, including both STEMI and NSTEMI, particularly within emergency and acute care settings. Recent multicenter prospective studies, including the ROMIAE trial, have shown that AI-ECG algorithms can diagnose acute MI in emergency settings with an AUC of 0.878 comparable to or exceeding the performance of established risk scores and clinical assessments. When integrated with traditional risk stratification tools, AI-ECG further enhances clinical decision-making and patient triage [46]. Prospective multicenter studies have demonstrated that AI-ECG achieves diagnostic accuracy for acute MI comparable to the HEART score and superior to the GRACE 2.0 score, high-sensitivity troponin. When combined with traditional risk scores, AI-ECG enhances risk stratification and net reclassification, supporting its role as a valuable adjunct in emergency departments for rapidly ruling in or ruling out acute MI and predicting 30-day major adverse cardiovascular events [64]. While AI-ECG is not intended to replace clinical judgment or guideline-based care, it serves as a valuable decision support tool that can enhance the detection and management of acute MI, particularly when used alongside clinical assessments and laboratory data.

### 4.5 AI-ECG in Atrial Fibrillation (AF)

AI is increasingly being applied to the diagnosis of AF through innovative approaches, primarily leveraging machine learning and deep learning techniques. A key application is AI-enhanced ECG analysis, where algorithms can accurately detect AF from both single-lead and standard 12-lead ECG recordings. Remarkably, some AI models have demonstrated the ability to predict future AF from ECGs recorded during sinus rhythm [65]. According to the 2023 guidelines from the ACC/AHA/HRS, automated algorithms including AI-enabled ECG are considered sufficiently reliable for assessing AF frequency, duration, and burden in patients with a known diagnosis. These tools can support rhythm monitoring and inform management decisions when used in conjunction with clinical evaluation [66].

### 4.6 AI-ECG in Valvular Heart Disease

AI-ECG is increasingly recognized as a promising tool for the detection and risk stratification of valvular heart disease (VHD), including conditions such as aortic stenosis (AS), aortic regurgitation (AR), mitral regurgitation (MR), tricuspid regurgitation (TR), and pulmonary regurgitation. Deep learning based AI-ECG models have demonstrated the ability to identify moderate-to-severe VHD with AUC values typically ranging from 0.77 to 0.88 for individual valve lesions, and exceeding 0.80 for composite detection, across both internal and external validation cohorts. These models can detect subtle ECG changes associated with structural valve abnormalities, often preceding the onset of clinical symptoms or overt ECG findings [67, 68]. AI-ECG has also demonstrated the ability to predict the future development of significant regurgitant valvular heart diseases. Individuals identified as high-risk by AI-ECG models exhibit substantially elevated hazard ratios for incident mitral, aortic, and TR. These predictive findings are often associated with early, subclinical chamber remodeling detectable on imaging [47, 68]. The integration of AI-ECG with complementary modalities, such as auscultation, has been shown to enhance diagnostic efficiency in the evaluation of valvular heart disease, enabling more accurate and timely identification of structural abnormalities.

### 4.7 AI-ECG in Cardiac Amyloid

AI-ECG is emerging as a valuable tool for the early detection of cardiac amyloid, including both transthyretin amyloidosis (ATTR) and light-chain amyloidosis (AL) subtypes. Deep learning models applied to standard 12-lead ECGs have demonstrated high diagnostic accuracy (AUC 0.85–0.91), often identifying disease months to years before clinical recognition or imaging abnormalities. These models can also be adapted for use with single-lead or 6-lead ECGs, enabling screening across diverse care settings [48, 69]. These models are capable of detecting subtle but disease-specific electrical patterns that often go unrecognized by human interpreters. This allows AI-ECG to overcome the limited sensitivity of conventional ECG criteria such as low QRS voltage which is observed in fewer than 40% of biopsy-confirmed cases [48, 70]. AI-ECG can serve as an effective pre-screening tool to identify individuals at elevated risk for cardiac amyloid, thereby enabling prioritization for confirmatory imaging such as echocardiography or nuclear scintigraphy and facilitating earlier diagnosis and initiation of treatment. Longitudinal analysis using AI-ECG may enable tracking of preclinical disease progression, offering the potential to support earlier clinical intervention and more proactive management strategies for such patients [71].

## 5. Current Challenges

### 5.1 High School Athlete Screening

The use of ECG screening in high school cardiovascular evaluations has been a subject of long-term debate and research. Previous guidelines from the AHA/ACC advocated for pre-participation screening of competitive athletes through history and physical examination. This is mainly guided by the AHA’s 14-point evaluation and it did not endorse mandatory ECG screening for all high school students or athletes. AHA has cited concerns regarding cost-effectiveness, diagnostic limitations, and resource demands [72]. However, based on ESC 2020 guidelines and ACC 2025 Scientific Statement From the AHA/ACC, evidence show that ECG enhances detection of ion channelopathies, accessory pathways, and many cardiomyopathies, increasing the sensitivity of the pre-participation screening for detection of potentially fatal cardiac conditions from <30.5% to 94% [73, 74]. Moreover, contemporary ECG interpretation criteria have led to substantial improvements in the sensitivity and specificity of screening ECGs and have reduced interobserver variation in interpretation for such cases [43, 75].

According to current criteria for ECG interpretation, most of the abnormalities identified (increased voltages suggestive for hypertrophy, early repolarization, AV block of first degree and type 1 second degree, bradycardia, right ventricular conduction delays) are benign or clinically insignificant and do not require further investigation. The proportion of ECG abnormalities suspicious for underlying cardiac abnormalities (mostly, inverted T-wave in lateral-inferior precordial leads, WPW pattern, ectopic beats of uncommon morphology) do not exceed the 5% of all ECG findings in a young Caucasian population. However, greater caution is needed in African American and Caribbean population, where proportion of ECG abnormalities may be larger [73, 76]. ECG screening conveys logistical challenges including the necessity for physician oversight, consistent interpretation standards, and sustainable financial support. Therefore, an effective ECG-inclusive PPE necessitates the involvement of appropriately trained clinicians in the interpretation of athletic ECGs and timely access to appropriate resources for downstream secondary evaluations, including cardiology consultation, to minimize potential harms attributable to unnecessary or prolonged restriction from sports [77].

At moment, the United States lacks a standardized national protocol for ECG screening in adolescents at difference of most European counties, but several community-based initiatives are locally driven, often emerging in response to high-profile cases of sudden cardiac death.

### 5.2 Asymptomatic Silent Atrial Fibrillation

Silent AF is a prevalent condition, comprising 10–40% of all AF cases with increased incidence among older adults and individuals with diabetes. A key diagnostic challenge lies in the limitations of standard 12-lead ECGs and brief rhythm strips, which often fail to capture paroxysmal or transient episodes, particularly in asymptomatic patients. While extended monitoring modalities such as Holter monitors, event recorders, implantable devices, and wearables enhance detection rates, the clinical relevance of short-duration or low-burden subclinical AF remains ambiguous and subject of further research. This uncertainty complicates decisions regarding stroke risk stratification and the appropriate threshold for initiating anticoagulation therapy [74]. The ACC/AHA/HRS/CHEST emphasize that while ECG remains essential for confirming AF, subclinical AF (SCAF) detected by devices often necessitates visual verification to avoid false positives caused by artifacts and unnecessary medical management that may involve anticoagulation. Currently, there is no consensus regarding the minimum duration or burden of SCAF that justifies anticoagulation therapy. Misinterpretation of ECG data may lead to unnecessary treatment or heightened patient anxiety [78].

## 6. Future Direction

The future of ECG interpretation is rapidly evolving through the convergence of AI, digital health platforms, and continuous monitoring technologies. AI algorithms now detect subtle waveform anomalies, stratify cardiovascular risk, and even predict disease onset before symptoms emerge. When paired with wearable ECG devices and remote monitoring systems, clinicians gain the ability to track cardiac rhythms in real time. This can transform ECG from a static clinical snapshot into a dynamic tool embedded in daily life. To ensure safe and effective adoption, we will need robust regulatory oversight, validation studies, and clinician training in the latest technology. Ultimately, ECG is destined to become not only a diagnostic mainstay but a transformative instrument in precision cardiovascular medicine. In a recent study, the DeepRhythmAI model demonstrated superior performance compared to human ECG technicians in the interpretation of ambulatory electrocardiograms (ECGs). The AI system showed greater sensitivity and markedly reduced false-negative rates for detecting clinically significant arrhythmias, achieving a negative predictive value exceeding 99.9%. While its application was associated with a higher frequency of false positives, the potential benefits are notable: by enabling direct-to-physician reporting, DeepRhythmAI may help minimize missed diagnoses, decrease reliance on technician review, and streamline the overall efficiency of ambulatory rhythm monitoring [79].

There are many studies currently ongoing to evaluate these technical advancements. As an example, the Realtime Diagnosis from AI-ECG-Guided Screening for Atrial Fibrillation with Long Follow-Up (REGAL) study is a pragmatic, randomized trial that is testing whether AI-identified high-risk patients can benefit from long-term AF monitoring using Apple Watch ECGs. This study can transform AF screening by integrating consumer wearables into routine care, potentially reducing stroke and cognitive decline [75]. It will be exciting to see the results of these ongoing AI-ECG trials, as their findings have the potential to transform how we practice medicine by enabling earlier detection and ultimately improved patient outcomes.

Lastly, Data privacy remains a critical concern in the development and deployment of AI-driven ECG technologies. As these systems often rely on large volumes of patient data, it is essential to ensure that information is securely stored, anonymized, and used in compliance with ethical and regulatory standards. Protecting patient confidentiality must be a foundational priority, especially as wearable devices and remote monitoring tools become more integrated into everyday clinical practice.

## 7. Conclusions

ECG remains a vital diagnostic tool in modern medicine largely appreciated by its rapid, noninvasive, and reproducible insights into cardiac and systemic conditions. Its structured interpretation enables clinicians to identify life-threatening arrhythmias, ischemic events, and conduction abnormalities, while also revealing structural and extracardiac influences on cardiac electrophysiology. The integration of AI, wearable technologies, and continuous monitoring is redefining the role of ECG in modern medicine from a static diagnostic tool to a dynamic, real-time health monitor. While early results from AI-ECG are promising, the safe and effective adoption of these innovations will require rigorous validation, regulatory oversight, and attention to ethical considerations.
